# Enhancing Malaria Research, Surveillance, and Control in Endemic Areas of Kenya and Ethiopia

**DOI:** 10.4269/ajtmh.21-1303

**Published:** 2022-10-13

**Authors:** John I. Githure, Delenasaw Yewhalaw, Harrysone Atieli, Elizabeth Hemming-Schroeder, Ming-Chieh Lee, Xiaoming Wang, Guofa Zhou, Daibin Zhong, Christopher L. King, Arlene Dent, Wolfgang Richard Mukabana, Teshome Degefa, Kuolin Hsu, Andrew K. Githeko, Gordon Okomo, Lilyana Dayo, Kora Tushune, Charles O. Omondi, Hiwot S. Taffese, James W. Kazura, Guiyun Yan

**Affiliations:** ^1^ Tom Mboya University College, Homa Bay, Kenya;; ^2^ Department of Medical Laboratory Sciences, Institute of Health, Jimma University, Jimma, Ethiopia;; ^3^ Tropical and Infectious Diseases Research Center, Jimma University, Jimma, Ethiopia;; ^4^ School of Public Health and Community Development, Maseno University, Kisumu, Kenya;; ^5^ Center for Global Health & Diseases, Case Western Reserve University, Cleveland, Ohio;; ^6^ Program in Public Health, University of California at Irvine, Irvine, California;; ^7^ Department of Biology, University of Nairobi, Nairobi, Kenya;; ^8^ Center for Hydrometeorology and Remote Sensing, Department of Civil and Environmental Engineering, University of California at Irvine, Irvine, California;; ^9^ Centre for Global Health Research, Kenya Medical Research Institute, Kisumu, Kenya;; ^10^ Ministry of Health, Homa Bay County, Homa Bay, Kenya;; ^11^ Ministry of Health, Kisumu County, Kisumu, Kenya;; ^12^ Department of Health Management and Policy, Faculty of Public Health, Jimma University, Jimma, Ethiopia;; ^13^ National Malaria Program, Federal Ministry of Health, Addis Ababa, Ethiopia

## Abstract

Malaria control programs in Africa encounter daunting challenges that hinder progressive steps toward elimination of the disease. These challenges include widespread insecticide resistance in mosquito vectors, increasing outdoor malaria transmission, lack of vector surveillance and control tools suitable for outdoor biting vectors, weakness in malaria surveillance, and an inadequate number of skilled healthcare personnel. Ecological and epidemiological changes induced by environmental modifications resulting from water resource development projects pose additional barriers to malaria control. Cognizant of these challenges, our International Center of Excellence for Malaria Research (ICEMR) works in close collaboration with relevant government ministries and agencies to align its research efforts with the objectives and strategies of the national malaria control and elimination programs for the benefit of local communities. Our overall goal is to assess the impact of water resource development projects, shifting agricultural practices, and vector interventions on *Plasmodium falciparum* and *P. vivax* malaria in Kenya and Ethiopia. From 2017 to date, the ICEMR has advanced knowledge of malaria epidemiology, transmission, immunology, and pathogenesis, and developed tools to enhance vector surveillance and control, improved clinical malaria surveillance and diagnostic methods, and strengthened the capacity of local healthcare providers. Research findings from the ICEMR will inform health policy and strategic planning by ministries of health in their quest to sustain malaria control and achieve elimination goals.

## INTRODUCTION

Malaria remains a significant public health concern in Kenya and Ethiopia. Three-quarters of the 48 million residents of Kenya and 52% of the 103 million residents of Ethiopia are at risk of malaria.^1^^,^^2^ Despite major progress in malaria control in the past two decades, malaria burden remains high in these two countries. For example, in 2020, there were an estimated 2.7 million and 4.2 million malaria cases in Kenya and Ethiopia, respectively.^3^ Malaria control strategies in both countries are deployed according to risk stratification based on routinely collected malaria case data from health facilities or from entomological surveillance.^1^^,^^2^ For example, Kenya is stratified into four epidemiological zones on the basis of malaria endemicity: Lake and Coastal endemic zones, where transmission is high throughout the year; seasonal transmission zones in arid and semiarid areas in Northern and Southeastern parts of the country, where there is a short period of intense transmission during the rainy season; malaria epidemic-prone areas of Western highlands; and a low-risk area in the central Kenyan highlands.^2^ Indoor residual spraying (IRS) of insecticides and intermittent presumptive treatment in pregnancy (IPTp) are added to the standard vector control intervention, long-lasting insecticidal-treated nets (LLINs).^4^ Similarly, Ethiopia is stratified into five broad strata according to annual parasite incidence: malaria-free, very low, low, moderate, and high transmission. Vector control methods are adjusted according to malaria risk strata.^5^

Kenya’s 2019–2023 Malaria Strategy aims to reduce the incidence and deaths due to malaria by 75% of 2016 levels by 2023 and to establish systems for malaria elimination in selected low-transmission counties.^2^ The 2020–2025 Ethiopian National Malaria Strategic Plan aims to reduce malaria morbidity and mortality by 50% from the 2020 baseline, achieve zero indigenous malaria cases in districts with low incidence, and eliminate malaria in the country by 2030.^5^ Achieving these ambitious goals must overcome many challenges. For example, insecticide resistance and outdoor malaria transmission are widely reported across Kenya and Ethiopia.^6^^–^^8^ Human-induced environmental modifications, such as deforestation, urbanization, and water resource development projects further alter vector ecology, malaria transmission dynamics, and disease risk.^9^^–^^11^ Changes in vector ecology and behaviors have limited the utility of currently available vector surveillance tools and the success of the existing first-line vector interventions, LLINs and IRS.^12^ Additionally, human migration associated with environmental modifications, natural disasters, or civil conflicts may introduce new parasite clones and drug resistant strains that confound elimination efforts.^13^

Increased knowledge of the impact of rapidly changing land use modifications and water resources and intensive malaria control measures on vector biology and malaria epidemiology is required to achieve the goals of national malaria control and elimination. In addition, new tools for surveillance and control of outdoor transmission are needed. Malaria risk is determined by complex interactions among biological factors (e.g., vector behavior, insecticide resistance, and malarial drug resistance), environmental factors (e.g., agriculture and irrigation), socioeconomic factors (financial cost of malaria interventions and access to high-quality healthcare), and operational factors such as implementation in local communities.^14^ Consequently, improving malaria intervention measures requires a multidisciplinary and multisectoral approach. Our International Center of Excellence for Malaria Research (ICEMR) has been conducting multidisciplinary research on malaria transmission, epidemiology, and immunology through a strong partnership with collaborators in Kenya and Ethiopia. In this article, we describe our ICEMR’s engagement with local and national partners and communities, collaborative research efforts with these stakeholders, and outcomes of these research activities.

## ENGAGEMENT OF PARTNERS AND COMMUNITIES

**Figure 1.**
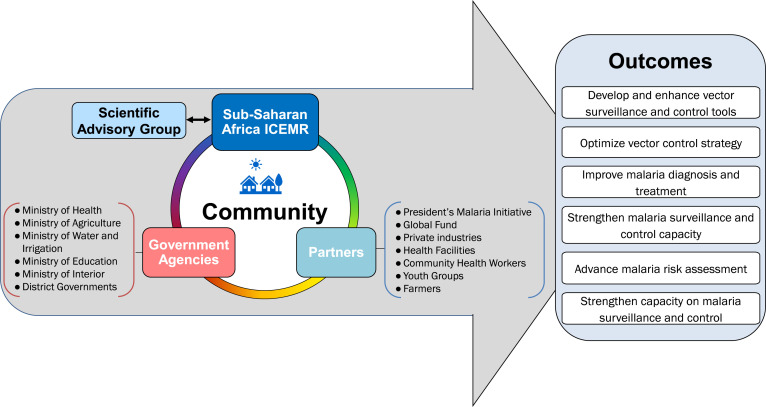
International Center of Excellence for Malaria Research (ICEMR) engagement with government agencies, academic and research institutions, partners, and local communities. Research studies are guided by the Scientific Advisory Group. The project has engaged Kenyan and Ethiopian government agencies and local and global partners. Outcomes of the project facilitate malaria surveillance and control in the community.

**Figure 2.**
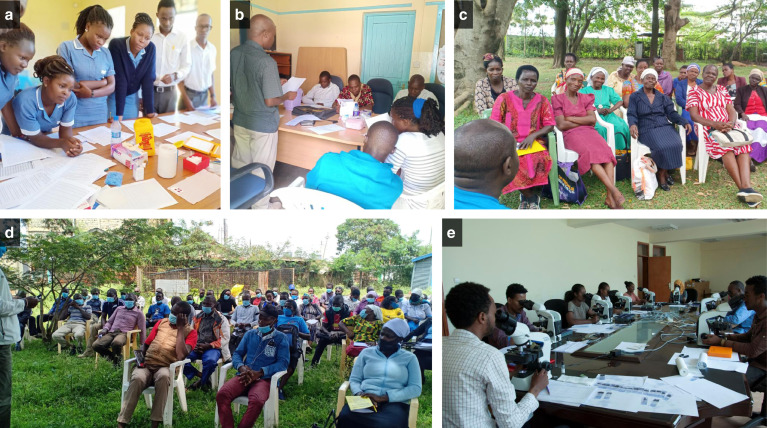
Community engagement in ICEMR activities in Kenya and Ethiopia: (**A**) health facility nurses receiving training in malaria passive surveillance; (**B**) meeting with hospital management team; (**C**) community health workers receiving training in active surveillance; (**D**) indoor residual spray training of community operators; and (**E**) malaria diagnosis training of ICEMR staff.

The main scientific aims of our ICEMR are to: 1) examine the impact of water resource development projects and irrigation-based agricultural activities on malaria risk and 2) generate high-quality quantitative data that can be used to enhance malaria control and elimination efforts in Kenya and Ethiopia. Research activities and progress made by the ICEMR have been reported annually to the Scientific Advisory Group appointed by the US National Institutes of Health (NIH) that provides advice and guidance in project implementation. Because the ICEMR addresses cross-disciplinary issues related to agriculture, water management, and human health, we have built a strong partnership with diverse government agencies (e.g., Ministries of Health [MoH], Agriculture, Interior, and Water and Irrigation) and academic and research institutions in Kenya and Ethiopia. Local communities are the beneficiaries of this interaction (Figure 1). We operationalized our ICEMR with the knowledge and support of national malaria control programs (NMCP) and implemented technology transfer at the county and community levels. Engagement of the ICEMR with these various partners is summarized in Table 1. At the national level, we have collaborated and consulted with MoHs, the President’s Malaria Initiative (PMI) of USAID, and the Global Fund to Fight AIDS, Tuberculosis, and Malaria, and contributed to national malaria control strategy development and policy recommendations concerned with different ecoepidemiological settings. At the county and local community levels, the ICEMR has contributed to strengthen the capacity and quality control of health facilities staff, malaria control program coordinators and managers, community health workers, and village youth groups. These interactions include a series of educational activities and technical trainings pertinent to malaria surveillance and control. Specific topics have included active case detection, passive case detection, reactive case detection, mass blood surveys, development and evaluation of vector surveillance and control tools, malaria awareness, and data collection, collation, and reporting. Attendees at these meetings and trainings included nurses, community health workers, and laboratory technicians at the various ICEMR study sites (Figure 2A–E). Our ICEMR also interacts with irrigation projects that are sponsored by local and national governments or privately owned. The Arjo Diddessa Sugar Factory in Ethiopia is sponsored by the government of Ethiopia while the Saudi Star Agricultural Development Company in Gambella is privately owned. The Kimira-Oluch Smallholder Farm Improvement Project in Homa Bay, Kenya, is sponsored by the national government. Hence, communities in both Kenya and Ethiopia have benefited from these interactions based on the following key outcomes: 1) developing and enhancing vector surveillance and control tools; 2) supporting in optimizing vector control strategies; 3) improving malaria diagnosis and treatment; 4) strengthening malaria surveillance and control capacity; 5) advancing malaria risk assessment; and 6) strengthening the capacity and sustainability of malaria surveillance and control.

**Table 1:** Engagement of ICEMR with partners in Kenya and Ethiopia

Partners	ICEMR engagement
Ministry of Health (MoH)	
National level President’s Malaria Initiative (PMI)/USAID Global Fund	MoH Vector Control Committee of Experts/Vector Control Working Group meetings National Malaria Technical Advisory Committee Pan-Africa Mosquito Control Association LLIN mass distribution IRS implementation strategy Contributing data to guide national malaria control strategy development and policy recommendations
County level Health Management Teams (CHMTs)	LLIN mass distribution IRS deployment Disseminated information and shared data with CHMTs
Health facilities	Nurses, malaria coordinators, and public health officers in data sharing Malaria surveillance, diagnosis, and case management Community awareness and social behavioral change communication
Community health workers (CHWs)	Active and passive case detection Community awareness Community case management Mobilization for vector control interventions
Ministry of Agriculture	
National Irrigation Authority Crop protection and livestock keeping	Irrigation scheme and water management Pesticide use and registration
Ministry of Water, Sanitation, and Irrigation	Shared information and data with stakeholders on impact of irrigation and dams on malaria risks Irrigation management
Ministry of Education	Primary school malaria awareness campaigns Promotion of malaria control
Ministry of Interior and Coordination of National Government	County commissioners, village chiefs and village elders in community mobilization and participation Malaria advocacy and control
Government-owned and private industries	
Arjo Diddessa Sugar Factory, Ethiopia Saudi Star Agricultural Development Company, Ethiopia Kimira-Oluch Smallholder Farm Improvement Project, Kenya	Mapping larval habitats and malaria risk Active and passive malaria surveillance Hotspot identification Community case management by CHW Guiding vector control Sharing malaria information
Academic and Research Institutions	
Jimma University, Addis Ababa University, Maseno University, Tom Mboya University, University of Nairobi Kenya Medical Research Institute, International Livestock Research Institute, National Council for Science, Technology and Innovation, Ethiopian Public Health Institute	Knowledge generation on malaria Research on malaria surveillance, control tools, and strategies Generate data and share findings with policy-makers Student and junior scientist training and research capacity building Facility and equipment sharing

ICEMR = International Center of Excellence for Malaria Research; IRS = indoor residual spraying; LLIN = lasting insecticidal-treated net.

## DEVELOPING AND ENHANCING MALARIA VECTOR SURVEILLANCE AND CONTROL TOOLS

### Vector surveillance tools.

Alternative and cost-effective tools suitable for outdoor vector surveillance are required to assess the extent and impact of outdoor malaria transmission. Though the human landing catch method is considered the gold standard for indoor and outdoor malaria vector surveillance, it has the drawbacks of difficulty in standardization, cost, and imposing malaria risk to the collectors. The ICEMR has improved three vector surveillance tools—the sticky clay pot trap, human odor-baited light trap, and human-baited double net/CDC light trap combination.^15^^,^^16^ The sticky clay pot trap adds sticky paper to a previously used clay pot trap^17^ to enhance mosquito collection efficacy. It is made of locally available materials, has low maintenance cost, is easy to use, and has a capacity of capturing 1.6 times more *Anopheles gambiae* mosquitoes compared with an ordinary clay pot.^16^ The human odor-baited CDC light trap and human-baited double net/CDC light trap combination caught two to three times and six times as many mosquitoes as a standard CDC light trap, respectively.^15^ The human-baited double-net/CDC light trap combination yielded a similar vector density as human landing catch.^18^ These exposure-free mosquito surveillance tools pose no risk of mosquito bites compared with human landing catches and, thus, are recommended for outdoor biting malaria vector surveillance and monitoring impact of vector control interventions.

### Long-lasting insecticidal-treated nets with piperonyl butoxide (PBO) synergist.

In 2021, the Kenya MoH introduced PBO nets as a pilot intervention in three counties in Western Kenya, where insecticide resistance is high and malaria prevalence is >20%.^19^ Our ICEMR is currently conducting an implementation study to evaluate the effectiveness of PBO nets and IRS on clinical malaria in comparison to conventional LLINs. Analysis of data from first 9 months of the study showed 43% reduction in transmission intensity and 48% reduction in clinical malaria incidence in villages that received PBO nets compared with those that received the standard pyrethroid only LLINs. Incidence reduction due to PBO nets was lower than Actellic 300CS-based IRS (adjusted incidence ratio 0.93); however, the cost of PBO nets was lower than IRS. The average cost per person protected is US $6.5 for PBO nets versus $8.4 for IRS in 2021, making PBO nets more cost-effective in terms of cost per malaria case averted. (Calculated as the annual program costs divided by the total number of cases averted, the total cost was $34 for PBO nets as compared with $55 for IRS.) Results from this study will provide information for rational decision-making with regard to the rollout of PBO nets in areas with pyrethroid-resistant malaria vectors.

### Larviciding.

Larval source management (LSM) is recommended by the World Health Organization (WHO) as a supplementary measure to core interventions in areas, where larval habitats are few, fixed, and findable. LSM may also be considered for use during specific times of the year when habitats are restricted, for example, the dry season. Larviciding has not been incorporated in the national control programs in Kenya and Ethiopia, but a few small-scale research trials of water-dispersible granular formulations of *Bacillus thuringienis* var. *israelensis* (*Bti*) and *B. sphaericus* (*Bs*) were conducted in Western and Coastal Kenya with significant reduction in adult mosquito densities.^20^^–^^23^ The main constraint with microbial larvicides used in these trials is their short duration of efficacy that would require frequent and costly application in NMCP. We previously evaluated the effectiveness of a slow-release *Bti* and *Bs* briquette formulation in Kisumu County where malaria transmission is perennial and high and Western Kenya highland areas, where malaria transmission is unstable and low. The findings demonstrated a significant reduction in indoor and outdoor biting density of malaria vectors and transmission intensity in both areas.^24^^,^^25^ These results will guide the national malaria control program of Kenya on LSM strategy, including the selection of microbial larvicide formulation, as they plan to implement larviciding using microbicides.

### Optimal combination of vector control tools.

Key malaria vector control tools—LLIN and IRS—have been very effective in Kenya and Ethiopia. However, their effectiveness has apparently diminished due to insecticide resistance and outdoor malaria transmission. On the other hand, IRS is labor intensive and expensive and LSM may not be cost-effective and applicable in all ecoepidemiological settings.^26^ Given the limitations of these tools, important questions faced by the malaria control managers need to be answered. For example, how should these vector control tools be combined and implemented to have synergy and maximal impact on malaria burden under the given funding constraints? Further, how should malaria control interventions be adapted locally to changing vector ecology, behavior, and malaria risks? Our ICEMR is conducting a cluster-randomized sequential, multiple assignment randomized trial in Kisumu County in Kenya to evaluate the impact of adaptive interventions that involve sequential and combinational use of PBO nets, IRS, and LSM for malaria control.^27^ Results from this study, anticipated to be complete in 2024, will provide a strategy for effective interventions tailored to local vector ecology, malaria risks, and cost effectiveness.

## IMPROVING MALARIA DIAGNOSIS AND TREATMENT

### Ultrasensitive malaria rapid diagnostic tests.

The WHO recommends that all malaria suspected cases should be treated only after confirmatory diagnosis by either microscopy or rapid diagnostic test (RDT).^28^ Although microscopic inspection of blood smears is the historical standard for *Plasmodium* parasite diagnosis, this method requires experienced slide readers to provide accurate diagnosis. The RDT, on the other hand, has benefits over microscopy in that it is less labor intensive, does not require electricity, and can be performed by unskilled personnel. However, RDT sensitivity can be compromised by the high prevalence of *Pfhrp-*2/3 gene deletion with false–negative results, especially in Ethiopia.^29^ The changing national malaria control landscape from control to elimination requires easy to use and sensitive diagnostic methods. The ICEMR evaluated the sensitivity and specificity of different malaria diagnostic methods at health facilities using microscopy, conventional CareStart RDT, and Alere ultrasensitive RDT. The results showed a 21% positivity rate by microscopy, 30% positivity by conventional RDT, and 36% positivity by ultrasensitive RDT. These results are indicative of ultrasensitive RDT detecting more malaria cases than the conventional RDT and microscopy. The finding from this study will help NMCP to consider using ultrasensitive RDT in areas approaching malaria elimination.

### *Plasmodium vivax* treatment with low-dose primaquine.

*Plasmodium vivax* endemicity is a major barrier to malaria elimination across much of Sub-Saharan Africa, including Ethiopia, where this *Plasmodium* species accounts for 35% of febrile malaria cases.^30^ Unlike *P. falciparum, P. vivax* has the ability to relapse weeks to years after exposure to infective mosquitoes because of its dormant liver or hypnozoite stage that are a potential source of relapsing blood-stage infections. Vivax relapses can only be prevented by eliminating hypnozoites, and primaquine (PQ) is currently the only licensed drug for radical cure. However, the effectiveness of a 3-day course of chloroquine and 14-day low-dose PQ combination treatment on relapse of *P. vivax* infections in a real world situation in Ethiopia is unknown. The ICEMR is currently conducting an observational study to examine the efficacy of PQ for preventing recurrence or relapse of *P. vivax* and the reduction of *P. vivax* infectiousness to mosquitoes in Ethiopia. We have observed that a large proportion of the Ethiopian study participants that received the chloroquine–PQ combination treatment showed recurrence of blood-stage *P. vivax* within the 6-month follow-up period. This study is in progress. Data to date indicate there is low adherence to completion of the 14-day PQ treatment regimen. Note that similar studies were not being conducted in the ICEMR Western Kenyan study sites because vivax malaria is very rare in this area of the country.

## STRENGTHENING MALARIA SURVEILLANCE

Malaria burden in the two ICEMR countries is monitored by routine data collected from health facilities and community surveillance that is aggregated and reported on a monthly basis in the electronic health information system such as the District Health Information System 2 (DHIS-2) in Kenya and electronic Health Management Information System (e-HMIS) in Ethiopia. These data are used for malaria risk stratification and malaria control strategy development. A number of factors can affect the quality of health facility data, including treatment seeking behavior by residents, diagnosis quality, quality and completeness of case reporting, and occasional nonoperation of health facilities due to staff strikes. The ICEMR has established demographic, entomological, and epidemiological surveillances in the study sites, tracked preventive measures, treatment seeking behavior, and population movement. This dataset has provided a robust and precise sampling framework for evaluating the impact of existing or new malaria control interventions. For example, the high malaria burden in many districts in Kenya and Ethiopia calls for improving the effectiveness of existing interventions. Optimizing first-line intervention tools and integrating newly approved or prequalified products into control programs should be based on precise information related to temporal changes in local malaria epidemiology and vector bionomics that can be obtained from such surveillance systems.^31^ Additionally, clinical malaria case incidence detected by active case detection in a cohort of residents in Kisumu County using the demographic surveillance system (DSS) database was found to be significantly higher than the incidence detected by active case detection. A healthcare seeking behavior survey indicated that a considerable proportion of residents did not seek diagnosis and treatment at health facilities; rather, community residents purchased antimalarial drugs from local drug stores (31.5%) or used herbal medicine (3.5%). We estimated that about 35% of community residents who experience clinical malaria are not captured in the DHIS-2. Under reporting of malaria, cases in the DHIS-2 will not reflect the true trend of malaria prevalence/incidence and assessment of impact of malaria interventions. This omission has implications pertinent to the reliability and completeness of the DHIS-2. Accordingly, data from online health information systems should be used with caution when extrapolated to population-level malaria risk and burden. These findings will guide the Kenyan MoH in strengthening HMIS by improved malaria advocacy, community awareness, and community case management of malaria.

## ADVANCING MALARIA RISK ASSESSMENT

Many African countries including Ethiopia and Kenya are developing irrigation schemes and hydroelectric power projects with the aim of ensuring food security and sustained energy production. Our studies indicate that communities in close proximity to irrigated areas and dams are at higher risk of *Plasmodium* infection compared with those living farther away due to increased mosquito proliferation resulting from environmental changes induced by these development projects. These data underscore the need to strike a balance among food security, economic development, and health services through enforcement of environmental and health impact assessments prior to construction of water resource projects. One of the research goals of our ICEMR is to develop a cost-effective and realistic plan to mitigate mosquito proliferation and malaria transmission before and after environmental modifications are implemented. This will require intersectoral collaboration and strengthening of healthcare delivery systems in communities located in and near water resource development projects. Integration of hydrological models with malaria transmission models, an approach adopted by the ICEMR, can assist the risk assessment and development of rational water-management strategies for malaria burden mitigation.^32^

## STRENGTHENING AND SUSTAINING CAPACITY ON MALARIA SURVEILLANCE AND CONTROL

One of the challenges faced by NMCP in Sub-Saharan Africa is heterogeneity of transmission in different settings whereby an intervention in a particular zone may not necessarily be effective in another zone. With the devolution of health services from the national to the county level, local capacity is critical to address community malaria surveillance and control needs. Unfortunately, most countries in Africa have an inadequate number of skilled personnel at the local level to manage and implement malaria surveillance and control activities. The ICEMR played a pivotal role in strengthening capacity of various health cadres in the national malaria control program. Nurses, technicians, malaria coordinators, public health officers, and health workers at the local community level have received training pertinent to malaria parasite and mosquito surveillance techniques, molecular techniques, genotyping, vector ecology, vector behavior, insecticide resistance monitoring, and evaluation of vector control interventions. Youth groups from the study sites have been trained to perform IRS, distribute LLINs, and collect and analyze healthcare data. The ICEMR has provided research training opportunities to 12 PhD and MSc students from Kenya and Ethiopia related to malaria vector biology, epidemiology, immunology, and pathogenesis. To sustain the capacity in malaria surveillance and control in both countries, training of graduate students and junior scientists is also being supported by grants from the NIH Fogarty International Center and local universities in Kenya and Ethiopia. This effort has strengthened malaria control and elimination at the national and district levels as these trainees are involved in designing, implementing, monitoring, and evaluating in malaria control and elimination program. Moreover, some of our ICEMR staffs serve as members of the malaria technical advisory group and national vector control working group in Kenya and Ethiopia.

## CONCLUSON

Changes in vector ecology, behavior, malaria risk, and insecticide resistance coupled with weak healthcare systems and increased costs of interventions are challenges to the success of malaria control programs in Africa. Environmental modifications that impact water use for irrigation undertaken to ensure food security and boost energy generation further complicate malaria control and elimination. Our ICEMR has been playing an important role in improving vector surveillance and control tools, enhancing malaria surveillance, developing optimal control strategies, and research capacity building in Kenya and Ethiopia. Through partnerships with MoH, other government agencies and nongovernmental organizations, the ICEMR has generated new evidence to inform local strategic planning for malaria control and elimination that will inform policy recommendations made by decision-makers and stakeholders in Kenya and Ethiopia.
